# Wound size and location affect the prognosis of penetrating ocular injury

**DOI:** 10.1186/s12886-023-03015-y

**Published:** 2023-06-07

**Authors:** Xue Gao, Qiuqiu Zhang, Fang Wang, Xuewei Li, Chunli Ma, Yixiao Li, Xiaofei Zhao, Han Zhang

**Affiliations:** 1 Department of Ophthalmology, The Second Hospital, Cheeloo College of Medicine, Shandong University, Jinan, 250033 China; 2 Department of Ophthalmology, Zaozhuang Municipal Hospital, Shandong Zaozhuang, 27710 China; 3 Department of Ophthalmology, Shandong Provincial Hospital Affiliated to Shandong First Medical University, Jinan, 250021 China; 4 Weifang Medical University, Shandong 261000 Weifang, P.R. China; 5 Shandong First Medical University and Shandong Academy of Medical Sciences, Jinan, 250117 China; 6 Department of Ophthalmology, Shandong Provincial Hospital, Cheeloo College of Medicine, Shandong University, Jinan, 250021 China

**Keywords:** Ocular trauma, Penetrating injury, Clinical characteristics, OTS, Zone analysis

## Abstract

**Background:**

Ocular trauma is a leading cause of vision loss. Penetrating ocular injury is a major type of open globe injury(OGI), while its epidemiology and clinical characteristics are still uncertain. The aim of this study is to reveal the prevalence and prognostic factors of penetrating ocular injury in the Shandong province.

**Methods:**

A retrospective study of penetrating ocular injury was performed at the Second Hospital of Shandong University, from January 2010 to December 2019. Demographic information, injury causes, ocular trauma types, and initial and final visual acuity(VA) were analyzed. To obtain more precise characteristics of penetrating injury, the eye global was divided into three zones and analyzed.

**Results:**

Among 210 OGI, there are 83 penetrating injuries which account for 39.5% of all. In addition, the final VA of 59 penetrating injuries recovered to 0.1 or better, which possesses the highest frequency among OGI. In order to research the relationship between the wound location and the final VA, we took 74 cases of penetrating injuries without retina or optic nerve damage for analysis. Results show that 62 were male and 12 were female. The average age was 36.01 ± 14.15. The most frequent occupation is the worker followed by the peasant. Statistics show that there is an obvious deviation in the Ocular trauma score (OTS) predicting the final VA and the actual final VA in the 45–65 score group (*p* < 0.05). Results suggest that the commonest penetrating injury zone is zone III (32 cases, 43.8%). Zone III, which is farthest from the center of the visual axis, has the largest improvement of the final VA (*p* = 0.0001). On the contrary, there is no statistical difference in the visual improvement in zone I and zone I + II that involves the injury of the central visual axis.

**Conclusion:**

This study describes the epidemiology and clinical characteristics of patients hospitalized for penetrating ocular injury without retina damage in Shandong province. It can be concluded that larger size and closer location to the visual axis of damage are accompanied by worse prognosis improvement. The study provides a better understanding of the disease and enlightenment for the prediction of visual prognosis.

## Background

Ocular trauma is a major cause of vision loss and blindness [1, 2]. Around the world, there are approximately 55 million patients suffering from ocular trauma each year, and about 1.6 million of them will result in blindness finally [3, 4]. Mechanical ocular trauma could be subdivided into open and closed globe injuries by the Birmingham Eye Trauma Terminology (BETT) [5–7]. OGI is a major cause of visual impairment among all kinds of ocular injuries [8–10]. Penetrating ocular injuries is a major type of OGI that can cause severe visual impairment [1, 11]. The epidemiological statistics of penetrating ocular trauma have been reported in developed countries such as the USA [3, 12–14], Europe [15] and Australia [16], and some developing countries including India, Iran, and Egypt [9–12]. Although the statistical data on ocular trauma in several southern provinces of China are available, the statistics on ocular trauma in China are still insufficient [1, 4, 17].

Assessment of visual functions of penetrating wounds is important for patient management, surgical operation, and the prediction of the prognosis of penetrating injury. Kuhn et al. [18] developed the prognostic OTS model to predict the visual outcome of patients after ocular trauma. The initial vision, eyeball rupture, endophthalmitis, eyeball perforation, retinal detachment, and presence/absence of relative afferent pupillary defects (RAPD) are identified as the main influencing factors. OTS is widely used to predict the prognosis by summing up the scores of the six factors mentioned above. Schmidt et al. [19] proposed another prognostic model, the classification and regression tree (CART), to predict visual function in patients after OGI. However, there are several limitations in those methods: both models have been proposed for more than 10 years; the size and location of the wound are ignored by those methods; some pathological factors or symptoms such as endophthalmitis and RAPD are difficult to evaluate at the early stage of ocular trauma [4].

Pure penetrating injury without retina and optic nerve damage usually results in better final VA [20], so the assessment of visual functions of wounds is important for patient management and the prediction of the prognosis of such diseases. Therefore, we collected and analyzed the data of penetrating ocular injury, in order to illuminate the relationship between the wound location and the final VA and provide a better understanding of the disease and enlightenment for the prediction of visual prognosis.

## Methods

The research was approved by the Ethics Committee of our Institute and followed the guidance of the Helsinki Declaration. Data were collected from all patients with a principal diagnosis of ocular trauma (ICD code: S05.600) admitted to the Department of Ophthalmology in the Second Hospital of Shandong University, Jinan, Shandong province, China, from January 2010 to December 2019. All cases of ocular trauma were diagnosed according to the International Classification of Diseases, Tenth Revision, Clinical Modification (ICD-10-CM). Ocular trauma cases were searched using ICD-10 code S05 in the electronic medical records.

### Aim

The aim of this study is to illuminate the prevalence and potential factors that affects the prognosis of patients with penetrating ocular injury in Shandong province.

### Study design and patients

Penetrating ocular injury is specifically divided into three zones by the location of the global injury in this study. The center of 3 mm in diameter of the cornea is defined as zone I, and the posterior 1.5 mm next to zone I is defined as zone II. Furthermore, the border of zone II to 6 mm posterior of the corneoscleral limbus is defined as zone III. The number of cases, initial VA, and final VA of each zone are collected and analyzed respectively. Ocular Trauma Score (OTS) is calculated to assess the extent of the globe injury. Final VA is obtained after the 6-month follow-up.

The OGI and closed-global injury are distinguished based on the BETT [16] and the degree of injury is classified according to Ocular Trauma Classification System (OTCS) [17]. The types of OGI are classified as penetrating injury, intraocular foreign body injury, perforating injury, and global rupture. Visual acuity is classified into five grades: NLP (no light perception), LP/ HM (light perception/ hand move),0.005–0.095, 0.1–0.4, and ≥ 0.5 [21].

Patients who meet the following criteria are included in this study. (1) Patients with penetrating ocular injury. (2) Patients who could comply with the vision examination. (3) Patients who did not suffer from any eye diseases before.

Meanwhile, patients who meet the following criteria are excluded from this study. (1) Patients who can not comply with the ophthalmic examination. (2) Patients who can not comply with the doctor's advice and follow-up. (3) Patients who have previous treatment history of eye disease. (4) Patients suffering trauma that affected the retina or optic nerve.

Details of all patients are collected from the medical records system including age, gender, occupational classification, place of residence, cause and location of ocular trauma, classification and distribution of ocular trauma, and initial and final VA. The final visual acuity recovered to 0.1 or better was defined as a success [8]. All the data are analyzed by grouping.

### Statistical analysis

Statistical analysis of all quantitative data including descriptive Statistical analyses were performed using SPSS (Version 27.0; IBM Corporation, Armonk, NY, USA). Demographics and patient characteristics are reported as percentages and cases in each group and include type of ocular trauma, distribution of age, initial VA, OTS predicted VA, corneal partition of the wound. *P* < 0.05 was considered a statistically significant difference.

Kruskal–Wallis test was used to compare differences in postoperative VA in patients with different types of eye trauma. The difference analysis of preoperative and postoperative VA in patients with penetrating eye injuries that do not injure the retina were performed using Wilcoxon signed-rank test. Wilcoxon signed-rank test was used to evaluate differences in OTS prediction of VA and actual final VA, the method was also been used to compare preoperative and postoperative VA improvement in different corneal partitions.

## Results

### Penetrating ocular injuries possess a high proportion and fine final VA in all open-globe injuries

A total of 210 open-globe injuries were hospitalized from 1 January 2010 to 31 December 2019 at the Second Hospital of Shandong University. Among the 210 open-globe injuries, as shown in Table 1, rupture injury demonstrated the highest frequency (119 cases, 56.7%) followed by penetrating injury (83 cases, 39.5%) and intraocular foreign body (IOFB) (8 cases, 3.8%). No perforating cases were exhibited in our study. It was noticeable that 59 cases of 83 penetrating injuries (71.1%) could improve their final VA to 0.1 or better, which was higher than rupture (9 cases, 7.6%) (*p* = 0.000, < 0.05). Due to the high proportion and fine final VA of penetrating injuries, this type of ocular injury was selected for further study.

**Table 1:** The number, ratio, frequency and percentage of final VA > 0.1of four types of OGI

Type	Frequency (Percentage%)	Final VA > 0.1 Frequency (Percentage%)
Penetrating	83 (39.5%)	59 (71.1%)
Perforating	0 (0%)	0 (0%)
Rupture	119 (56.7%)	9 (7.6%)
Intraocular foreign body	8 (3.8%)	3 (37.5%)

### The final VA penetrating injuries improved significantly compared with the initial VA

The frequency and the percentage of the final VA and initial VA of penetrating injuries were shown in Table 2. There was a significantly different distribution between the initial VA and final VA (*p* = 0.0001, *p* < 0.05). LP/HM accounted for the highest proportion in initial VA (30 cases, 40.54%). After medical treatment, the proportion of LP/HM in final VA was reduced to 6.76% (5 cases), and a large proportion of patients demonstrated a best-corrected final VA exceeding 0.1(59 cases, 79.73%). Moreover, 41 cases of those patients could improve the final VA to 0.5. It could be concluded that the final VA penetrating injuries improved significantly compared with the initial VA.

**Table 2:** Comparison and correlation between initial and final VA of the 74 penetrating injuries

	Initial		Final	
Grade of VA	Frequency	Percentage (%)	Frequency	Percentage (%)
NLP	1	1.35	1	1.35
LP-HM	30	40.54	5	6.76
0.005–0.095	18	24.32	9	12.16
0.1–0.4	16	21.62	18	24.32
≥ 0.5	9	12.16	41	55.41

*NLP* no light perception, *LP* light perception, *HM* hand movement

### Demographics and characteristics of hospitalized penetrating ocular injuries over 10 years period

Then, the demographics and characteristics of penetrating injuries without optic nerve and retina damage were analyzed (Table 3). Among 74 patients taken into our study, there were no binocular injury case in our study, and no significant difference in laterality with a ratio of 44.6:55.4 between the left and right eyes (Table 3). The ratio between the male and female patients attains 5.17:1, namely, 62 (83.8%) were male and 12 (16.2%) were female. The average age was 36.01 ± 14.15. The age distribution showed that the peak occurrence of ocular trauma occurred in the 21–30 age group (21 cases, 28.4%), followed by the 31–40 age group (20 cases, 27%), 41–50 age group (11 cases, 14.9%) and 51–60 age group (11 cases, 14.9%) in sequence. Of these 74 patients with penetrating ocular injury, the most common occupation was worker (48 cases, 64.86%), followed by peasant (19 cases, 25.28%). Ocular trauma occurred more frequently in rural areas (42 cases, 57%) than in urban areas (32 cases, 43%). Most of the penetrating injuries occurred during working (54 cases, 72.97%).

**Table 3:** Demographics and characteristics of patients hospitalized for penetrating injuries

Total patients	N(%)
Laterality of eyes(%)	
OD	33 (44.6)
OS	41 (55.4)
OU	0
Gender(%)	
Male	62 (83.8)
Female	12 (16.2)
1–10	3 (4)
11–20	5 (6.8)
21–30	21 (28.4)
31–40	20 (27)
41–50	11 (14.9)
51–60	11 (14.9)
60–90	3 (4)
Occupation(%)	
Peasant	19 (25.68)
Worker	48 (64.86)
Student	4 (5.4)
Child	2 (2.7)
Other	2 (2.7)
Location(%)	
Rural	42 (56.76)
Urban	32 (43.24)
Settings(%)	
Home	4 (5.4)
Work	60 (81)
MVA (motor vehicles and automobile)	2 (2.7)
Assault	5 (6.8)
Other	3 (4)

### The final VA predicted by OTS had a deviation compared with the actual final VA in the OTS score 45–65 group at the last follow-up

To examine whether OTS could be applied to the VA prediction of penetrating ocular injury, we compared the OTS-predicted final VA with the actual final VA.

Results of statistical analysis showed that OTS predicted final VA was consistent with actual VA in the OTS score 66–80 group (*p* = 0.108, *p* > 0.05) and 81–91 group (*p* = 1, *p* > 0.05) respectively. But there was a large deviation in the prediction of the OTS score 45–65 group, with 29 cases, 39.2% of all patients (*p* = 0.001, *p* < 0.05). In this group, final VA predicted by the OTS method were NLP (28%), LP-HM (26%), 0.005–0.095 (18%), 0.1–0.4 (13%), and > 0.5 (15%) respectively, while the actual final VA were NLP (3.4%), LP-HM (3.8%), 0.005–0.095(24.1%), 0.1–0.4(13.8%) and ≥ 0.5(44.8%) respectively. There are no patients in the 0–44 group and 92–100 group, so the two groups couldn’t be analyzed (Table 4).

**Table 4:** Comparison of the predicted VA based on the OTS versus the final VA

Raw score sum	OTS category	number	NLP(%)	LP/HM(%)	0.05–0.95(%)	0.1–0.4(%)	≥ 0.5(%)
0–44	1	0	73/0	17/0	7/0	2/0	1/0
45–65	2	29	28/3.4	26/3.8	18/24.1	13/13.8	15/44.8
66–80	3	36	2/0	11/2.78	15/8.33	28/30.56	44/58.33
81–91	4	9	1/0	2/0	2/0	21/22.2	74/77.8
92–100	5	0	0/0	1/0	2/0	5/0	92/0

The former percent value refers to the predicted VA based on the OTS, and the latter percent value refers to the final VA at the last follow-up

### Larger size and closer location to the visual axis of damage accompany worse improvement

To better understand the character of penetrating ocular injury, we divided the anterior ocular into three zones in this study specifically. The center of 3 mm in diameter of the cornea was defined as zone I, and the posterior 1.5 mm next to zone I was defined as zone II. Furthermore, the border of zone II to 6 mm posterior of the corneoscleral limbus was defined as zone III.

**Fig. 1:**
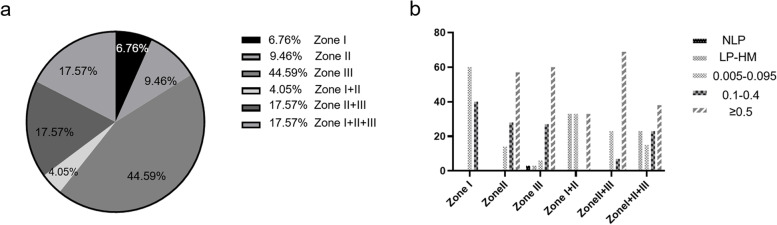
Distribution of penetrating ocular injuries and final visual acuity. **a** Distribution of penetrating ocular injuries in each zone; **b** Distribution of final visual acuity in different zones of 74 penetrating ocular injuries

Results show that zone III was the commonest site being wounded (33 cases, 44.6%), followed by zone II + III (13 cases, 17.6%), zone I + II + III (13 cases, 17.6%), zone II (7 cases, 9.5%), zone I (5 cases, 6.8%) and zone I + II (3 cases, 4%) (Fig. 1a).

The distribution of final VA in different zones of all cases was shown in Fig. 1b. Furthermore, we analyzed the final VA after therapy better than 0.1 and defined it as success. As shown in Table 5, wound location in zone I (5/5 = 100%) owned the highest rate of success, followed by zone III (30/33 = 90.9%), zone II (6/7 = 85.7%), zone II + III (6/13 = 46%), and zone I + II + III (8/13 = 61.5%), while the zone with the worst final VA was zone I + II (1/3 = 33.3%). However, statistical results of VA improvement showed that there were statistically significant between the initial VA and the final VA in Zone II (*p* = 0.003), zone III (*p* = 0.0001), zone II + III (*p* = 0.016), and zone I + II + III (*p* = 0.0004) (Table 5). On the contrary, there was no statistical difference in the visual improvement in zone I and zone I + II that involves the injury of the central visual axis. It could be concluded that zone III, the commonest site being wounded, had the largest improvement of final VA. Larger size and closer location to the visual axis of damage accompany worse improvement.

**Table 5:** The distribution of the initial and final VA in different zones of penetrating ocular injuries

	Zone I		Zone II		Zone III		Zone I + II		Zone II + III		Zone I + II + III	
Grade of VA	Initial	Final	Initial	Final	Initial	Final	Initial	Final	Initial	Final	Initial	Final
NLP	0	0	0	0	1	1	0	0	0	0	0	0
LP-HM	2	0	3	0	10	1	2	1	4	0	9	3
0.005–0.095	1	0	4	1	7	2	0	1	3	3	3	2
0.1–0.4	2	3	0	2	9	9	1	0	3	1	1	3
≥ 0.5	0	2	0	4	6	20	0	1	3	9	0	5

## Discussion

The penetrating injury is a common type of OGI [1, 11]. This retrospective study presents data concerning the prevalence and clinical characteristics of patients who were hospitalized for penetrating ocular injuries in the second hospital of Shandong University.

### Penetrating ocular injury is important in OGI

According to the results, we found that penetrating ocular injuries possess a high proportion (83 cases, 39.5%) and highest proportion (59 cases, 71.1%) of final VA > 0.1 among OGI (*p* < 0.05) (Table 1). This is also in accord with previous studies which reported that penetrating injuries usually preserved better final VA than IOFB injuries after medical treatment [4]. Due to the full-thickness injury of the cornea or sclera induced by blunt trauma, rupture injuries usually trigger severe complications and result in a worse prognosis than penetrating injuries. Since IOFB injuries are often accompanied by endophthalmitis and retinal damage after ocular trauma, the final VA of IOFB injuries is usually worse than penetrating injuries.

Due to the high proportion and fine final VA, penetrating injuries without retina and optic nerve damage are selected for further research with the aim to provide a better understanding of the disease and enlightenment for the prediction of visual prognosis.

### Demographics

Our study demonstrates that age and gender are strongly associated with the incidence of penetrating injury. The rates of penetrating injury were higher in patients ranging from the 21–40 age group, especially in males. These findings are consistent with other studies which reported that ocular trauma often occurred in the dominant labor force ranging from 30–50 years old in China [17, 22, 23].

As involved in our study, the workplace is the most frequent place of injury. The incidence of work-related ocular trauma (54 cases, 72.97%) revealed in our research is conform to results from previous research, which reported the range of work-related ocular trauma is from 25.4% to 73.7% [24–26]. Since more and more peasants turning into migrant workers, the incidence of penetrating injuries may increase furthermore [1]. The main contributing factor to the higher proportion of work-related ocular trauma is the workers’ lack of safety awareness. Therefore, safety awareness must be cultivated, and workers must be educated to effectively protect their eyes during work [17, 27, 28].

### The final VA of penetrating injuries predicted by OTS had obvious deviation compared with the actual final VA in OTS score 45–65 group

We compared the OTS-predicted final VA with the actual final VA to evaluate the accuracy of the OTS method. Of the three groups being analyzed, OTS predicting final VA is consistent with actual VA in the OTS score 66–80 group and 81–91 group respectively, but there is a large deviation in 45–65 group (*p* = 0.001, *p* < 0.05). The OTS method is not accurate when used to evaluate the final VA of penetrating injury. As reported in Korean research, the positive predictive value of OTS in OGI is as low as 75.3% [8, 29]. The following limitations may contribute to the deviation. (1) The OTS method has been more than 20 years since it was developed. (2) Some factors are difficult to describe such as RAPD, especially for OGI patients with pain and scare brought by the ocular trauma. (3) The location and size of the wound which are regarded as important factors in descript the final VA of OGI were not considered in the OTS method [4].

### The final VA of penetrating injury related to the wound size and location

To better understand the character of penetrating ocular injury, we divided the anterior ocular into three zones in this study specifically. Zone III was the most popular affected area in our study. Besides the largest area among the three zones, escape instinct is a possible reason that most injuries are away from the center of the visual axis.

The success rates of the single zone (zones I, II, and III) are 100%, 85.7%, and 90.9% respectively. Slight astigmatism triggered by the small size of the scar located in one single zone may attribute to the final VA outcome mentioned above.

However, the success rates in patients with extended injury in zone I + II, II + III, and zone I + II + III are 33.3%, 46%, and 61.5% respectively, which is worse than that in a single zone. In terms of patients involved in zone I + II, the proportion of success is the lowest (33.3%) of all the zones. Because the injuries involved in multi-zone are usually large, the visual axis is frequently blocked by large corneal scars, and the VA is often affected by severe astigmatism triggered by large scars.

Statistical analysis shows that there are significant differences between the initial VA and the final VA in zone II, zone III, zone II + III, and zone I + II + III. Since the location of zone I is close to the visual axis, the affection of the scar to the transparency of the central corneal area and astigmatism near the central area may contribute to the weak improvement of injuries involved in zone I. However, for the injury in zone I + II + III which has a relatively large wound, astigmatism near the central area can be ameliorated by adjusting the position and tightness of the suture. It may account for the improvement of final VA in zone I + II + III while the detailed reason requires further investigations.

Overall, we demonstrated that the final VA of penetrating injury is usually related to the wound size and location. Larger size and closer location to the visual axis of damage accompany a worse prognosis.

### Shortcomings

In order to study the effect of simple corneoscleral injury on visual acuity, we excluded cases with retinal or optic nerve damage, vitreous hemorrhage, endophthalmitis, so the number of cases taking into statistical analysis ultimately is not abundant enough.

Since the injury site of ocular trauma involves wide range, we systematically divided the patient's injury range into different zones and analysis the visual acuity of different zones. Due to the detailed corneal divisions, the cases falling into each zone is a little small, so the sensitivity and specificity of OTS score could not be statistically analyzed.

In the further study, we intent to cooperate with other hospitals to enlarge the sample size and verify our conclusion about the prognosis of penetrating ocular injury in this manuscript.

## Conclusion

This study describes the epidemiology and clinical characteristics of patients hospitalized for penetrating ocular injury without retina and optic nerve damage in Shandong province. Penetrating ocular injuries possess a high proportion and better final VA among all types of OGI. Age, gender, and profession are strongly associated with the incidence of penetrating injury. Moreover, larger size and closer location to the visual axis of damage accompany the worse prognosis. The epidemiological investigation and detailed division of penetrating ocular injury provide a better understanding of the disease and enlightenment for the prediction of visual prognosis.

## Data Availability

The datasets supporting the conclusions of this article are included within the article.

## Abbreviations

BETT: Birmingham Eye Trauma Terminology
CART: Classification and regression tree
IOFB: Intraocular foreign body
LP/ HM: Light perception/ hand move
NLP: No light perception
OGI: Open-globe injury
OTCS: Ocular Trauma Classification System
OTS: Ocular trauma score
RAPD: Relative afferent pupillary defects
VA: Visual acuity
